# Using field-evolved resistance to Cry1F maize in a lepidopteran pest to demonstrate no adverse effects of Cry1F on one of its major predators

**DOI:** 10.1007/s11248-012-9604-4

**Published:** 2012-02-29

**Authors:** Jun-Ce Tian, Hilda L. Collins, Jörg Romeis, Steven E. Naranjo, Richard L. Hellmich, Anthony M. Shelton

**Affiliations:** 1 Department of Entomology, Cornell University/New York State Agricultural Experiment Station (NYSAES), 630 West North Street, Geneva, NY 14456 USA; 2 Agroscope Reckenholz-Tänikon Research Station ART, Reckenholzstr. 191, 8046 Zurich, Switzerland; 3 USDA-ARS, Arid Land Agricultural Research Center, 21881 North Cardon Lane, Maricopa, AZ 85138 USA; 4 Department of Entomology, USDA–ARS, Corn Insects and Crop Genetics Research Unit, Iowa State University, 110 Genetics Laboratory c/o Insectary, Ames, IA 50011 USA

**Keywords:** Cry1F, Biosafety, *Coleomegilla maculata*, *Spodoptera frugiperda*

## Abstract

*Spodoptera frugiperda* (JE Smith) represents the first documented case of field-evolved resistance to a genetically engineered crop expressing an insecticidal protein from *Bacillus thuringiensis* (Bt). In this case it was Cry1F-expressing maize (Mycogen 2A517). The ladybird beetle, *Coleomegilla maculata*, is a common and abundant predator that suppresses pest populations in maize and many other cropping systems. Its larvae and adults are polyphagous, feeding on aphids, thrips, lepidopteran eggs and larvae, as well as plant tissues. Thus, *C. maculata* may be exposed to Bt proteins expressed in genetically engineered crops by several pathways. Using Cry1F-resistant *S. frugiperda* larvae as prey, we evaluated the potential impact of Cry1F-expressing maize on several fitness parameters of *C. maculata* over two generations. Using Cry1F resistant prey removed any potential prey-mediated effects. Duration of larval and pupal stages, adult weight and female fecundity of *C. maculata* were not different when they were fed resistant *S. frugiperda* larvae reared on either Bt or control maize leaves during both generations. ELISA and insect-sensitive bioassays showed *C. maculata* were exposed to bioactive Cry1F protein. The insecticidal protein had no effect on *C. maculata* larvae, even though larvae contained 20–32 ng of Cry1F/g by fresh weight. Over all, our results demonstrated that the Cry1F protein did not affect important fitness parameters of one of *S. frugiperda*’s major predators and that Cry1F protein did not accumulate but was strongly diluted when transferred during trophic interactions.

## Introduction

Maize, *Zea mays* L., is one of the most important world crops with over 150 million hectares planted in 2009 (FAOSTAT 2009). Because many important pests of maize are lepidopterans, transgenic maize expressing insecticidal proteins (Cry toxins) from *Bacillus thuringiensis* (Bt maize) has been commercially grown in the United States and globally since 1996. Bt maize has been adopted to control a series of stalk, whorl, leaf and ear infesting Lepidoptera (Hellmich et al. 2008) and globally was planted in 16 countries on a total of 39 million hectares in 2010 (James 2010). Although Bt crops have been planted for 15 years and the vast majority of published reports have shown no negative effects of Bt crops on non-target organisms (Romeis et al. 2006; Marvier et al. 2007; Wolfenbarger et al. 2008; Naranjo 2009), the ecological safety of Bt plants continues to be debated. Much of this debate has focused non-target beneficial species (predators and parasitoids) and on whether any purported negative effects are in fact due to the Bt protein or quality of the host or prey on which the natural enemy feeds (Shelton et al. 2009a).

The fall armyworm (FAW), *Spodoptera frugiperda* (J. E. Smith) (Lepidoptera: Noctuidae), is an agricultural pest of tropical-subtropical origin in the Western Hemisphere. Its larvae feed on more then 60 plant species; however, maize, peanuts, rice, cotton and sorghum are favored (Luginbill 1928). FAW often infest whorl stage maize where it can substantially reduce plant growth, but the insect also infests ears where it can cause considerable damage. Although estimates of damage are difficult to assess, in the late 1970 s losses in Georgia alone were estimated at $137 million (Sparks 1979). Insecticidal control to prevent injury in field maize is difficult and generally not cost effective. The first Bt-maize plants grown in the United States expressed Cry1Ab proteins that were primarily targeted against the European corn borer, *Ostrinia nubilalis* (Hübner) (Leidoptera: Crambidae) but were less effective against FAW. In 2001, Herculex I^®^ (Cry1F) maize was approved in the United States and targeted both *O. nubilalis* and FAW (Hellmich et al. 2008). Reports indicated that it could substantially reduce losses by FAW (Buntin et al. 2004; Buntin 2008; Siebert et al. 2008). However, FAW resistance to Herculex I^®^ maize was documented in Puerto Rico by 2006, only 3 years after its commercialization (Matten et al. 2008; Tabashnik et al. 2009; Storer et al. 2010). The high levels of field damage combined with a high laboratory-derived resistance ratio made FAW resistance the first well-documented case of field-evolved resistance to Bt plants. This resistance also afforded us an opportunity to use it for studies on tritrophic interactions with natural enemies of FAW.

One major ecological concern regarding the biosafety of Bt crops on the environment is their potential effects on non-target organisms (NTO), especially predators and parasitoids that play an important role in pest control (Romeis et al. 2006; Kennedy 2008; Romeis et al. 2008). The ladybird beetle *Coleomegilla maculata* (DeGeer) (Coleoptera: Coccinellidae) is a common and abundant predator that suppresses pest populations in maize and many cropping systems. Its larvae and adults are polyphagous, feeding on aphids, thrips, lepidopteran eggs and larvae, as well as plant tissues. Thus, *C. maculata* may be exposed by several pathways to insecticidal proteins expressed in Bt crops. Because *C. maculata* is an important biological control agent and suitable for laboratory experiments, it is commonly used to evaluate the risks of Bt crops.

In research conducted on the potential effects of Bt maize (expressing Cry3Bb1 or Cry1Ab) and Bt cotton (expressing Cry2Ab and Cry1Ac) on *C. maculata*, no direct negative impacts by these Bt crops have been detected (Pilcher et al. 1997; Duan et al. 2002; Lundgren and Wiedenmann 2002, 2005; Li et al. 2011b). However, none of these studies were conducted with insects that had evolved resistance in the field to a Bt plant. Using Cry1F-resistant FAW from the field would allow one to avoid questions of potential differences in laboratory or field-derived Bt resistance as well as overcome any potential prey-mediated effects (Romeis et al. 2011) of Cry1F maize on *C. maculata*, thus providing additional assurance about its safety to this important predator. The objective of this study was to study the potential effects of Cry1F maize on *C. maculata* using Cry1F-resistant FAW as prey. Survival, development time, adult weight and female fecundity were evaluated over two generations.

## Materials and methods

### Plants

Seeds of Bt maize (Mycogen 2A517), expressing Cry1F protein, and the corresponding non-transformed near isoline (Mycogen 2A496) were obtained from Dow AgroSciences (Indianapolis, IN). Bt maize and non-Bt maize were grown simultaneously in the same green house at Cornell’s New York State Agricultural Experiment Station in Geneva, NY. Plants were grown in Ray Leach Cone-tainer Cells (diameter 3.8 cm; depth 21 cm; volume 164 ml) (Stuewe & Sons, Tangent, OR) at 21 ± 3°C under a light and dark regime of 16:8 h.

### Insects

A Cry1F-resistant strain of FAW was obtained from Dow AgroSciences in 2010 and maintained in our laboratory on artificial diet. This strain developed resistance to Cry1F maize in Puerto Rico (Storer et al. 2010) and is able to survive on Cry1F maize.

To detect the bioactivity of Cry1F, we used a susceptible strain of the diamondback moth, *Plutella xylostella* (Linnaeus) (Lepidoptera: Plutellidae), which has been continuously reared on artificial diet since 1988 (Shelton et al. 1991). Second instar *P. xylostella* were used for detecting bioactivity of Cry1F, as described below.

*Coleomegilla maculata* reared on artificial diet (Li et al. 2011a) were used in the tests. This colony originated from Pioneer Hi-Bred International, Inc. (Johnston, IA) and was maintained in a climatic chamber at 27 ± 1°C, 50 ± 10% RH, and 16:8 h photoperiod. Newly hatched 1st instar larvae were used.

### Expression of Cry1F in maize leaves

When maize reached the V1, V3, V5, V7, V10 and VT stage (Ritchie et al. 1992), three leaf samples were collected from both Bt and non-Bt maize. Each sample was approximately 20 mg and obtained from the second new leaf. All samples were weighed, put into 1.5 ml centrifuge tubes, and stored at −20°C until Cry1F levels were measured.

### Bioassay with *S. frugiperda*

FAW neonates were individually kept in 30-ml cups and fed leaves of Bt maize or non-Bt maize at the 5–8 leaf stage. There were 30 replications for both treatments. Leaves were changed daily and larvae checked daily until death or pupation. The number of days until pupation or death was recorded. After FAW reached pupation, pupal weight was recorded. The longevity of adults, not provided with any nourishment, also was recorded.

### Tri-trophic bioassay with *C. maculata*

First instar *C. maculata* were individually kept in 30-ml cups and supplied with either 1st or 2nd instar FAW fed Cry1F maize (V5) or control maize (V5). A piece of control maize leaf was placed in each cup to eliminate any problem with predators that also ingest leaf tissue. In addition, a water-saturated cotton ball was provided on the bottom of each cup to maintain humidity. FAW were changed daily and *C. maculata* were checked twice per day (9 a.m. and 9 p.m.), and the following parameters were recorded: survival and developmental time of larvae and pupae. In addition, newly emerged *C. maculata* adults were weighed. The experiment was initiated with 50 *C. maculata* larvae for each treatment. For assessing fecundity, 10 pairs of newly emerged *C. maculata* adults from both treatments were kept in individual Petri dishes (diameter 9 cm) and allowed to mate. Adults were fed shrimp eggs and agar solution for 20 days, according to the procedures of Li et al. (2011a). Eggs of *C. maculata* were removed and recorded daily. To investigate egg-hatching rates, 30 egg masses from both treatments were randomly selected and put into individual Petri dishes (diameter 9 cm) and monitored until eggs hatched.

The offspring (F2 of *C. maculata*) underwent another generation of testing, as described above.

### Cry1F residue in insects

Another 100 1st instar *C. maculata* for each treatment were reared as described for the tri-trophic bioassay. Three samples (6–10 insects as one replicate) from each treatment were collected when *C. maculata* reached the 2nd instar, 3rd instar, 4th instar, pupa and early adult stage. Newly hatched FAW, which were fed Cry1F maize (V5) or control maize (V5), were also sampled (10 larvae as one replicate, three replications) at 24, 48 and 72 h. The Cry1F toxin concentrations and bioactivities in the samples were determined by ELISA and bioactivity bioassays using *P. xylostella* larvae (see below).

### ELISA measurement

The concentrations of Cry1F in maize leaves and insects were measured by ELISA using Cry1F detection kits from Agdia (Elkhart, IN). Prior to analysis, all insects were washed with PBST buffer four times to remove any Bt toxin from the surface. Maize leaf samples were diluted at a rate of 1:20 (mg sample:μl PBST buffer) and fully ground by mortar and pestle. Insect samples were diluted at a rate of at least 1:10 (mg sample: μl PBST buffer) in 1.5 ml centrifuge tubes, and ground by hand using a plastic pestle. ELISA was performed according to the manufacturer’s instructions.

### Bioactivity of Cry1F after ingestion by FAW and *C. maculata*

Samples of Cry1F maize (V5) leaves, non-Bt maize (V5) leaves, FAW fed on Cry1F maize for 48 h, FAW fed on non-Bt maize for 48 h, 4th instar *C. maculata* fed on Cry1F maize-fed FAW and 4th instar *C. maculata* fed on non-Bt maize-fed FAW were used in this experiment. Cry1F containing samples were diluted to 4 ng Cry1F/ml. Corresponding control samples were diluted at a similar rate. Bond-spreader sticker (Loveland Industry, Loveland CO) was added at 0.1% to each sample solution before being applied to cabbage leaf disks (diameter 3 cm). Ten 2nd instar Cry1F-susceptive *P. xylostella* (strain G88) were placed on each of the leaf disks inside 30 ml Cometware™ plastic cups (WNA, Covington, KY) with 5 replicates per treatment. Larval mortality was checked after 72 h at 27 ± 1°C.

### Statistical analyses

Data on ELISA and toxicity of Cry1F in plant tissue and insects were analyzed using one-way analysis of variance (ANOVA) and Tukey’s multiple-range test. Survival analysis of FAW fed on Bt maize or non-Bt maize was conducted using the Wilcoxon test for homogeneity. Data on life table parameters of FAW and *C. maculata* were analyzed using Student’s *t* test. Data on bioactivity of Cry1F were analyzed using one-way ANOVA and Tukey’s multiple comparison test. Before analysis, all percentage data were arcsine of square root transformed, as necessary, but untransformed means are presented. All statistical calculations were performed with SAS version 9.1 package (SAS Institute 2001). For all tests, α = 0.05.

## Results

### Cry1F in Bt maize, FAW and *C. maculata*

The Cry1F maize variety used in the present study was shown to express Cry1F at levels ranging from 2.38 to 4.33 μg/g fresh weight (FW) (Table 1). The Cry1F maize variety reached the highest Cry1F expression level at V3 stage. Cry1F expression level decreased as maize aged.

**Table 1:** Cry1F concentration in Cry1F maize leaves, *S. frugiperda* (FAW) and *Coleomagilla maculata* (n = 3)

Cry1F maize (μg/g FW)		FAW (ng/g FW)		*C. maculata* (ng/g FW)	
V1	3.98 ± 0.052 ab	Fed for 24 h	95.9 ± 9.03 b	2nd instar	20.3 ± 2.36 a
V3	4.33 ± 0.298 a	Fed for 48 h	211.8 ± 19.12 a	3rd instar	20.9 ± 3.36 a
V5	3.21 ± 0.233 ab	Fed for 72 h	176.3 ± 9.58 a	4th instar	32.2 ± 11.44 a
V7	2.64 ± 0.553 ab			Pupae	9.3 ± 1.35 a
V10	2.47 ± 0.531 b			Adults	Not detectable
VT	2.38 ± 0.257 b				

Mean (±SE) followed by different letters in the same column are significantly different (One-way ANOVA, *P* < 0.05)

*FW* fresh weight

Cry1F residue in FAW was at levels ranging from 95.9 to 211.8 ng/g FW (Table 1). The Cry1F concentrations in FAW that fed on Cry1F maize for 48 and 72 h were significantly higher than those in FAW that fed on Cry1F maize for 24 h.

The Cry1F concentrations in *C. maculata* larvae were 3–10 times lower than those in FAW, ranging from 20.3 to 32.2 ng/g FW (Table 1). Cry1F in *C. maculata* pupae was 9.3 ng/g. No Cry1F was detected in newly emerged *C. maculata* adults. As expected, no Cry1F was detected in non-Bt maize or FAW fed on non-Bt maize and *C. maculata* fed on non-Bt maize-fed FAW.

### Bioassay with FAW

There were no significant differences in survival of the FAW larvae when fed either Cry1F maize or control maize, and in both cases survival was >93% (*χ* ^2^ = 0.0014, *df* = 1, *P* = 0.97) (Table 2). A slight, but statistically significant difference, in total development time from larva to pupa occurred, but this amounted to only 0.6 days out of 17 days total (*t* = 2.5468, *df* = 55, *P* = 0.0137). There were no significant differences in any of the other life table parameters (Table 2).

**Table 2:** Impact of Cry1F maize on life table parameters of Cry1F-resistant *S. frugiperda*

Parameters	Cry1F maize	Non-Bt isoline
Survival (%)	96.67 a	93.33 a
Development time (days)		
Larva-pupation	17.5 ± 0.13 (29) a	16.9 ± 0.18 (28) b
Pupal stage	9.4 ± 0.24 (29) a	9.7 ± 0.25 (28) a
Larva-adult	26.9 ± 0.35 (29) a	26.5 ± 0.40 (28) a
Adult longevity (days)	4.6 ± 0.17 (29) a	4.7 ± 0.18 (28) a
Pupal weight (mg)	223.2 ± 4.3 (29) a	221.3 ± 3.6 (28) a

Mean (±SE) followed by different letters in the same row are significantly different (Survival: Wilcoxon test, *P* < 0.05; other parameters: Student’s *t* test, *P* < 0.05)

n, sample size

### Tri-trophic bioassay with *C. maculata*

After feeding on Bt maize-fed and non-Bt maize-fed FAW, *C. maculata* had three molts before they reached the pupal stage (Table 3). Adults emerged from pupae after 2.5–3.5 days pupation. There were no significant differences detected for any life table parameters of *C. maculata* between the Cry1F maize treatment and control maize treatment.

**Table 3:** Tri-trophic effects on life table parameters of *C. maculata* when fed Cry1F-resistant *S. frugiperda* larvae that were reared on Cry1F or non-Bt isoline maize leaves

Parameters	Cry1F maize	Non-Bt isoline
Development time (days)		
1st instar	3.16 ± 0.11 (31) a	3.23 ± 0.14 (28) a
2nd instar	2.81 ± 0.14 (29) a	2.98 ± 0.12 (28) a
3rd instar	3.32 ± 0.13 (28) a	2.98 ± 0.14 (28) a
4th instar	5.00 ± 0.12 (28) a	4.98 ± 0.11 (28) a
Pupal stage	3.18 ± 0.05 (28) a	3.19 ± 0.04 (28) a
Larvae-adults	17.43 ± 0.24 (28) a	17.38 ± 0.13 (28) a
Male fresh weight (mg)	7.83 ± 0.20 (15) a	7.89 ± 0.32 (13) a
Female fresh weight (mg)	10.12 ± 0.30 (13) a	9.86 ± 0.20 (15) a
Total fecundity	77.7 ± 16.2 (10) a	70.9 ± 17.0 (10) a
Egg hatching rate (%)	63.9 ± 3.76 (30) a	66.7 ± 2.77 (30) a

Mean (±SE) followed by different letters in the same row are significantly different (Student’s *t* test, *P* < 0.05)

n, sample size

Similar results were found for the second generation (Table 4). No significant differences were found for any life table parameters between the Cry1F maize treatment and control maize treatment.

**Table 4:** Tri-trophic effects of Cry1F maize on the progeny of *C. maculata* fed Cry1F-resistant *S. frugiperda* larvae that were reared on Cry1F or non-Bt isoline maize leaves

Parameters	Cry1F maize	Non-Bt isoline
Development time (days)		
1st instar	3.19 ± 0.17 (31) a	3.25 ± 0.17 (32) a
2nd instar	2.79 ± 0.16 (29) a	2.95 ± 0.20 (29) a
3rd instar	3.29 ± 0.14 (29) a	2.93 ± 0.20 (28) a
4th instar	5.16 ± 0.12 (29) a	5.11 ± 0.22 (28) a
Pupal stage	3.07 ± 0.05 (29) a	3.23 ± 0.05 (28) a
Larvae-adults	17.40 ± 0.20 (29) a	17.30 ± 0.34 (28) a
Male fresh weight (mg)	8.18 ± 0.12 (16) a	7.99 ± 0.19 (16) a
Female fresh weight (mg)	9.87 ± 0.15 (13) a	9.95 ± 0.24 (13) a
Total fecundity	74.0 ± 13.3 (10) a	58.5 ± 15.0 (10) a
Egg hatching rate (%)	70.4 ± 5.66 (30) a	73.0 ± 3.65 (30) a

Mean (±SE) followed by different letters in the same row are significantly different (Student’s *t* test, *P* < 0.05)

n, sample size

### Bioactivity of Cry1F after ingestion by FAW and *C. maculata*

Extracts from Cry1F maize leaves and Cry1F maize-fed FAW larvae were toxic to susceptible *P. xylostella* (Table 5). This indicates that *C. maculata* that fed on Cry1F maize-fed FAW were exposed to active Cry1F. However, Cry1F in *C. maculata* showed no toxicity to susceptible *P. xylostella*.

**Table 5:** Bioactivity of Cry1F residue from *S. frugiperda* and *C. maculata* to Cry1F-susceptible *Plutella xylostella* larvae

Treatment	Mortality % (Means ± SE)
Cry1F maize leaf	66.0 ± 9.27 a
Non-Bt maize leaf	10.0 ± 3.16 b
FAW reared on Cry1F maize leaf for 48 h	48.0 ± 4.90 a
FAW reared on non-Bt maize leaf for 48 h	6.0 ± 4.0 b
4th instar *C. maculata* fed on Cry1F maize-fed FAW	24.0 ± 2.45 b
4th instar *C. maculata* fed on non-Bt maize-fed FAW	18.0 ± 3.74 b
dH_2_O (Control)	8.0 ± 3.74 b

A total of 50 susceptible *P. xylostella* larvae were used in each treatment with 5 replications (10 larvae/replication). Means (±SE) followed by different letters are significantly different (One-way ANOVA, *P* < 0.05)

## Discussion

Bt crops, as one important integrated pest management (IPM) component, have reduced traditional insecticides use, providing benefits for the environment, economy and human health (Shelton et al. 2002; Brookes and Barfoot 2009; Naranjo 2011). However, there is a concern as to whether Bt crops are compatible with another IPM component, biological control. Biological control of insect pest through predators and parasitoids is an important element of IPM and so many studies have focused on potential effects of Bt crops on natural enemies (Romeis et al. 2008; Wolfenbarger et al. 2008; Naranjo 2009). Although the majority of studies have shown no negative effects of Bt crops on natural enemies, some laboratory studies have mistakenly measured the effects of prey or host quality and not the direct effect of Bt toxicity, e.g. Lövei et al. (2009). These effects have erroneously been interpreted as direct toxic effects of the Cry protein (Shelton et al. 2009a, b). Using Bt-resistant herbivores has been suggested as a way of overcoming the potential effects of prey/host-quality in an assessment of the effects of plant-expressed insecticidal proteins on natural enemies (Romeis et al. 2011).

A Bt-resistant strain eliminates effects of prey or host quality and also contains a higher concentration of Bt proteins when compared with a susceptible strain (Lawo et al. 2010). In this study, we used Cry1F-resistant FAW, the first herbivore to evolve resistance to a Bt plant in the field, as the carrier of Cry1F. Although others have used Bt resistant strains to overcome host quality effects (e.g. Li et al. 2011b), no other studies have used strains that have evolved resistance to a Bt plant under field conditions, thus adding another level of assurance. Our results showed that FAW feeding on Cry1F maize only contained 3–6% of the Cry1F proteins in the plant, and that there were no significant differences in survivorship or the development time from larva to adult stage compared to when they fed on non-Bt maize. This is consistent with the data in Storer et al. (2010) that indicated the resistant strain is unaffected by high concentrations of Cry1F. The bioactivity assays confirmed that Cry1F protein was still biologically active even after ingestion by FAW. Thus, with our Cry1F maize/Cry1F-resistant FAW/natural enemy (*C. maculata*) tri-trophic bioassay system, we were able to overcome any host quality effect and evaluate the direct potential toxicity of Cry1F to *C. maculata* through a biologically realistic pathway.

Our results demonstrated Cry1F maize had no significant impact on developmental time, adults weight and fecundity of *C. maculata*. This is consistent with another study that evaluated the potential effects of Bt crops on ladybird beetles. Lundgren and Wiedenmann (2002) and Duan et al. (2002) demonstrated Cry3Bb1 did not impact any of the fitness parameters (including the duration of larval and pupal stages, pupal weight, adult mobility, adult survivorship, and female fecundity) of *C. maculata* when the ladybird beetles were fed with Bt-maize pollen. Larval survival and development, adult survival, and adult dry weight did not differ for ladybird beetles, *Stethorus punctillum* (Weise) (Coleoptera: Coccinellidae), fed with spider mites, *Tetranychus urticae* (Koch) (Acari: Tetranychidae), reared on Cry3Bb1 maize or non-Bt maize (Li and Romeis 2010). Similarly, *C. maculata* survival, development time, adult weight and fecundity were not different when they were fed with resistant cabbage looper, *Trichoplusia ni* (Hübner) (Lepidoptera: Noctuidae), larvae reared on either Bt cotton (expressing Cry1Ac and Cry2Ab) or control cotton (Li et al. 2011b).

Cry1F maize leaves used in this study contained 2.7–3.4 μg/g FW Cry1F protein. Similar results of Cry1F protein levels in Bt maize leaves were reported in two other Cry1F maize varieties Herculex^®^ I (111 ng/g total protein) and Herculex^™^ I (10–23 ng/mg dry weight) (US Environmental Protection Agency Office of Pesticide Programs 2005; DuPont 2011). Our ELISA measurement demonstrated that only 10–20% of the Cry1F found in FAW larvae was detected in *C. maculata* larvae (Table 1). Similar dilution effects have been reported in other tritrophic studies. For example, when *C. maculata* was fed with Cry1Ac and Cry2Ab-expressing Bt cotton-fed cabbage looper, *T. ni*, Bt protein levels in predators were 21 times lower for Cry2Ab and 6 times lower for Cry1Ac compared to the concentrations in the prey (Li et al. 2011b). Similarly, when spider mites, *T. urticae*, fed with Cry3Bb1 maize were used as prey, the Cry3Bb1 protein level in larvae and adults of ladybird beetles, *S. punctillum* were 6 and 20 times lower than Bt protein concentrations measured in spider mites, respectively (Li and Romeis 2010). First and second instars of *Adalia bipunctata* (Linnaeus) contained 7–12 times lower levels of Bt proteins compared to the prey *T. urticae* that fed on Cry1Ac or Cry3Bb1-expressing Bt maize (Alvarez-Alfageme et al. 2011). This indicates that Bt protein did not bioaccumulate and biomagnify when transferred from prey to predator. Furthermore, bioactivity of Cry1F tests showed Cry1F protein in *C. maculata* was not bioactive (Table 5). We assume Cry1F decomposed in this predator to protein fragments that had no bioactivity.

To date, most studies have been conducted to assess the potential effects of Bt on NTOs for only a single generation. However, there has been some concern about whether adverse effects might only be manifested in subsequent generations. Our study indicated Bt maize did not harm *C. maculata* even when they were exposed to Cry1F for two generations. This helps support the 3-year field observation that Cry1F maize did not impact the abundance of ladybird beetles (including eggs, egg clutches, larvae and adults) (Higgins et al. 2009).

In conclusion, our studies with Cry 1F-resistant FAW, the first insect to have evolved resistance to a Bt plant in the field, allowed us to eliminate any potential prey-quality effects when examining the potential effect of Cry1F in a tri-trophic test with an important predator of FAW, *C. maculata*. Our studies demonstrated that Cry1F did not affect important fitness parameters of *C. maculata* and that Cry1F protein did not accumulate but rather decomposed when transferred during trophic interactions. These results, together with other published literature, demonstrate no adverse effects of Cry1F, and thus Cry1F-expressing Bt maize, on *C. maculata*.
